# Functional Crypto-Adenylate Cyclases Operate in Complex Plant Proteins

**DOI:** 10.3389/fpls.2021.711749

**Published:** 2021-08-12

**Authors:** Inas Al-Younis, Basem Moosa, Mateusz Kwiatkowski, Krzysztof Jaworski, Aloysius Wong, Chris Gehring

**Affiliations:** ^1^Biological and Environmental Sciences and Engineering Division, King Abdullah University of Science and Technology, Thuwal, Saudi Arabia; ^2^Physical Science and Engineering Division, King Abdullah University of Science and Technology, Thuwal, Saudi Arabia; ^3^Chair of Plant Physiology and Biotechnology, Faculty of Biological and Veterinary Sciences, Nicolaus Copernicus University in Toruń, Toruń, Poland; ^4^Department of Biology, College of Science and Technology, Wenzhou-Kean University, Wenzhou, China; ^5^Zhejiang Bioinformatics International Science and Technology Cooperation Center of Wenzhou-Kean University, Wenzhou, China; ^6^Department of Chemistry, Biology & Biotechnology, University of Perugia, Perugia, Italy

**Keywords:** adenylyl cyclase, 3',5'-cyclic adenosine monophosphate, crypto-enzymes, multi-domain moonlighting enzymes, catalytic center, *Arabidopsis thaliana*, 9-*cis*-epoxycarotenoid 11,12 dioxygenase

## Abstract

Adenylyl cyclases (ACs) and their catalytic product cAMP are regulatory components of many plant responses. Here, we show that an amino acid search motif based on annotated adenylate cyclases (ACs) identifies 12 unique *Arabidopsis thaliana* candidate ACs, four of which have a role in the biosynthesis of the stress hormone abscisic acid (ABA). One of these, the 9-cis-epoxycarotenoid dioxygenase (NCED3 and At3g14440), was identified by sequence and structural analysis as a putative AC and then tested experimentally with two different methods. Given that the *in vitro* activity is low (fmoles cAMP pmol^−1^ protein min^−1^), but highly reproducible, we term the enzyme a crypto-AC. Our results are consistent with a role for ACs with low activities in multi-domain moonlighting proteins that have at least one other distinct molecular function, such as catalysis or ion channel activation. We propose that crypto-ACs be examined from the perspective that considers their low activities as an innate feature of regulatory ACs embedded within multi-domain moonlighting proteins. It is therefore conceivable that crypto-ACs form integral components of complex plant proteins participating in intra-molecular regulatory mechanisms, and in this case, potentially linking cAMP to ABA synthesis.

## Introduction

In plants, cyclic nucleotide monophosphates and their cyclases are gaining increasing attention due to their involvement in crucial developmental and physiological processes, including regulating guard cell movements and responses to abiotic and biotic stresses (Gehring and Turek, 2017). Furthermore, the *Arabidopsis thaliana* cyclic nucleotide interactome harbors proteins that cross talk with cyclic nucleotides, nitric oxide (NO), and other reactive oxygen species to signal for plant defense responses (Donaldson et al., 2016). One of the cyclic nucleotide monophosphates, 3',5'-cyclic adenosine monophosphate (cAMP), has been shown to be involved in important biological processes, such as the regulation of cellular Ca^2+^ and K^+^ fluxes (Kurosaki and Nishi, 1993; Li et al., 1994; Talke et al., 2003; Lemtiri-Chlieh and Berkowitz, 2004; Ali et al., 2007). Today, cAMP is known to modulate key developmental processes, such as pollen tube growth and reorientation (Moutinho et al., 2001; Tsuruhara and Tezuka, 2001), promotion of cell division (Ehsan et al., 1998; Sabetta et al., 2016) and seed germination (Uematsu et al., 2007), and plant responses to biotic and abiotic stresses through the regulation of stomatal opening and defense-related genes (Jin and Wu, 1999; Ma et al., 2009; Sabetta et al., 2019; Blanco et al., 2020). Yet, plant enzymes that generate cAMP and adenylyl cyclases (ACs) were until recently unknown because homologs from bacteria and animal systems appeared to be absent in higher plants. The implementation of an amino acid search motif supported by structural modeling strategy (Wong et al., 2018; Zhou et al., 2021) has enabled the discovery of many AC domains. Many of those ACs are somewhat hidden in complex multi-functional proteins (Al-Younis et al., 2015; Chatukuta et al., 2018; Bianchet et al., 2019; Ruzvidzo et al., 2019). Although they reside in relatively small regions of proteins with well-defined primary functions, e.g., ion channels and phosphodiesterases (Kwiatkowski et al., 2021), their activities have been experimentally validated, while their molecular and biological significance are also increasingly being elucidated (Al-Younis et al., 2018; Chatukuta et al., 2018; Bianchet et al., 2019; Yang et al., 2021).

Given the possibility that many plant ACs remain undetected, we have used a novel motif-based approach to identify candidate ACs (Wong et al., 2018). This motif is built based on conserved amino acids in catalytic centers of canonical ACs from organisms across species (Ludidi and Gehring, 2003) and has since identified numerous nucleotide cyclases, including ACs (for review, see Gehring and Turek, 2017). Since the motif extracts only conserved amino acids that have direct function in catalysis at the catalytic center, candidate ACs identified by this approach are structurally dissimilar to canonical ACs as they assume the conformations of their primary domains, such as kinases and ion channels. Their activities seem also uncharacteristically but consistently lower than canonical stand-alone ACs, presumably due to the spatially more restricted function of the AC moonlighting sites in complex proteins (Turek and Irving, 2021). We propose to classify such enzymes as crypto-ACs. Incidentally, the same motif- and structure-based identification method has also successfully identified novel nitric oxide (NO) (Mulaudzi et al., 2011; Zarban et al., 2019; Wong et al., 2020a, 2021) and ABA binding sites (Ooi et al., 2017) in *Arabidopsis* proteins that perform regulatory roles much like the crypto-ACs.

Here, we queried the *Arabidopsis thaliana* proteome with a stringent AC catalytic center motif and identified a number of candidate ACs of which one, a 9-*cis*-epoxycarotenoid dioxygenase (NCED3 and At3g14440), was selected for computational and functional analyses since NCEDs are crucial components of ABA biosynthesis (Meier et al., 2011) and hence also plant stress responses. We propose that this AC detection search method can identify hidden ACs in other complex proteins and that this will in turn contribute to our understanding of cyclic nucleotide-dependent signaling in plants and other organisms.

## Results and Discussion

### Identification of Hidden Candidate ACs

**Figure 1:**
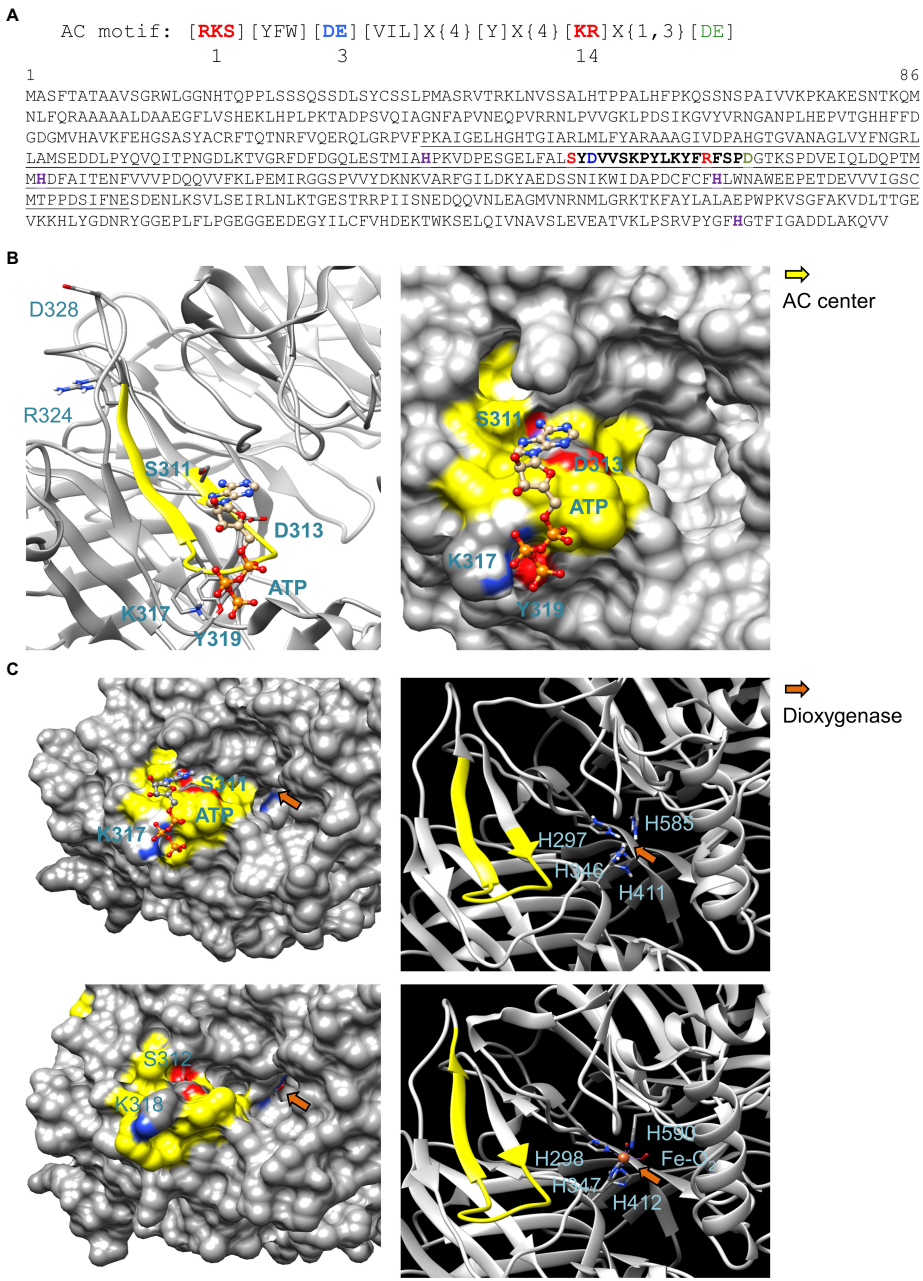
Sequence and structural analysis of 9-*cis*-epoxycarotenoid dioxygenase AtNCED3. **(A)** AC search motif and amino acid sequence of AtNCED3. The AC center motif (S311 – R324) is bolded in the amino acid sequence where position 1 (red) forms hydrogen bond with adenine, position 3 (blue) confers substrate specificity (D or E for ATP), and position 14 stabilizes the transition (ATP/cAMP). Amino acid for Mg^2+^/Mn^2+^ binding is labeled green. In the motif specific for ACs, the [DE] in position 3 (blue) allows for ATP binding. Amino acids in the square brackets denote amino acids allowed in this position, “X” denotes any amino acid, and curly brackets ({}) denotes the number of undetermined amino acids. Underlined amino acids indicate the AtNCED3 fragment that was cloned and expressed for functional studies *in vitro*. The histidine residues marked in magenta are required for the octahedral binding of Fe^2+^ which is required for dioxygenase activity. **(B)** Ribbon and surface models of AtNCED3 AC center docked with ATP. The AC catalytic center is highlighted in yellow, and key amino acids in the motif (except for D328 and R324) and amino acids that may interact with ATP are labeled, colored according to their charges, and shown as individual atoms in the ribbon models. AtNCED3 structure was modeled against the crystal structure of a maize viviparous14 protein (PDB ID: 3NPE). **(C)** Comparison of the AC and dioxygenase domains in AtNCED3 (top) and ZmNCED3 (bottom). AtNCED3 docked with ATP is represented as a surface model (left) with its AC and iron-binding histidine amino acids at the dioxygenase domain represented as a ribbon model (right) in the top panel with corresponding regions in the ZmNCED3 structure shown in the bottom panel. AC center is highlighted in yellow, and key amino acids in the motif are labeled in bold and colored according to their charges in the surface models. The iron-binding histidine residues in the dioxygenase domain (orange arrows) are labeled in the ribbon models.

We assembled a 14-amino acid long AC catalytic center motif containing the key amino acids that are conserved and have direct roles in substrate binding and/or catalysis (Figure 1A). These amino acids are separated by gaps as determined in the alignment of catalytic centers of annotated ACs in diverse prokaryotic and eukaryotic organisms. Notably, this AC search motif and its derivatives have successfully identified similar and experimentally confirmed ACs (Gehring, 2010; Ruzvidzo et al., 2019). When the *Arabidopsis thaliana* proteome was queried with this search term, it returned 12 proteins (Supplementary Table 1) one of which, a clathrin assembly protein (CLAP and At1g68110), has been shown to have AC activity (Chatukuta et al., 2018). We also noted significant enrichments in several gene ontology terms (GO) notably “biosynthetic process” (GO:0009058; *p* = 0.0064, FDA = 0.046). We selected AtNCED3 (At3g14440) for further analysis since it has (11',12') 9-*cis*-epoxycarotenoid cleavage activity and catalyzes the first step of ABA biosynthesis from carotenoids and in doing so, enabling plant response to water stress (Iuchi et al., 2001; Ruggiero et al., 2004; Endo et al., 2008; Frey et al., 2012). AtNCED3 is therefore a promising candidate for research that examines the link between plant stress, ABA, and cAMP.

### Structural Analysis of AtNCED3 AC and NCED3 Domains

Since the structure of AtNCED3 is yet to be determined, we built a model for AtNCED3 using homology modeling approach (Wong and Gehring, 2013) to analyze the AC and NCED3 domains. AtNCED3 was modeled against the crystal structure of a maize NCED3 (ZmNCED3) which is a key enzyme in the biosynthesis of the phytohormone ABA (PDB ID: 3NPE). At 69% identity and covering 86% of AtNCED3 amino acid sequence, ZmNCED3 was the best template option identified using the BLASTp tool available at https://blast.ncbi.nlm.nih.gov/Blast.cgi?PAGE=Proteins (Altschul et al., 1990) that compared AtNCED3 against a database of protein crystal structures in the protein databank (PDB). Based on the model, the AC center motif [RKS][YFW][DE][VIL]X{4}[Y]X(4)[KR]X{1,3}[DE] appears between S311 to R324 with the predicted cation-binding amino acid D328 located four residues downstream of the catalytic center (Figure 1A). Notably, the AC prediction tool ACPred available at http://gcpred.com/acpred/main.php (Xu et al., 2018) also identified with high confidence this same region as a candidate AC.

The secondary structure of the AC center assumes a slightly different fold compared to other experimentally validated AC centers that contain a helix-loop secondary fold (Figure 1B) (Al-Younis et al., 2015, 2018). The AC center occupies a distinct pocket that docks ATP with a mean binding affinity of −4.64 ± 0.03 kcal/mol calculated from a total of 10 positive docking solutions predicted by AutoDock Vina (Supplementary Figure 1). ATP assumes an orientation much like in experimentally validated AC centers (Al-Younis et al., 2015; Al-Younis et al., 2018; Ruzvidzo et al., 2019) where the adenine of ATP points toward position 1, which occupies the interior of the AC pocket, and the phosphate which points toward position 14, which occupies the solvent-exposed entrance area of the AC pocket (Figures 1B,C). We refer this substrate orientation as a “correct binding pose” (Wong et al., 2018). However, spatial evaluation of its binding pose suggests that the phosphate end of ATP could interact more feasibly with K317 rather than R324 which, together with the predicted metal coordinating D328 residue, is seemingly too distant for interactions with ATP. Instead, the aromatic ring of a tyrosine (Y319) that is located two amino acids downstream of K317 could offer metal ion coordination (Figure 1B). Since binding pose of ATP to ACs identified through this motif has been previously ascertained (Chatukuta et al., 2018; Ruzvidzo et al., 2019), we analyzed all docking solutions across two independent simulations to determine the positive binding pose frequency which in this case is 55.6%, thus lending confidence to the prediction that this protein can function as an AC. Docking clusters and data, and representative structures are presented in Supplementary Figure 1.

AtNCED3 AC does not resemble canonical ACs since the motif only includes the key amino acids at the catalytic centers of conventional ACs. Moreover, ACs identified by this motif approach occupy moonlighting sites that are separate from primary domains that range from kinases to transporters and gas-sensing regions (Al-Younis et al., 2015, 2018; Wheeler et al., 2017; Wong et al., 2020b). Due to these diverse structural architectures, these ACs assume structural folds that do not resemble the conventional classes of ACs which are often stand-alone proteins (Xu et al., 2018; Su et al., 2019). Thus, while structural evaluation supports favorable binding with ATP which is a pre-requisite for catalysis, there were also structural features that are unique to the AC center of NCED3.

### Experimental Validation of AtNCED3 AC Activity

**Figure 2:**
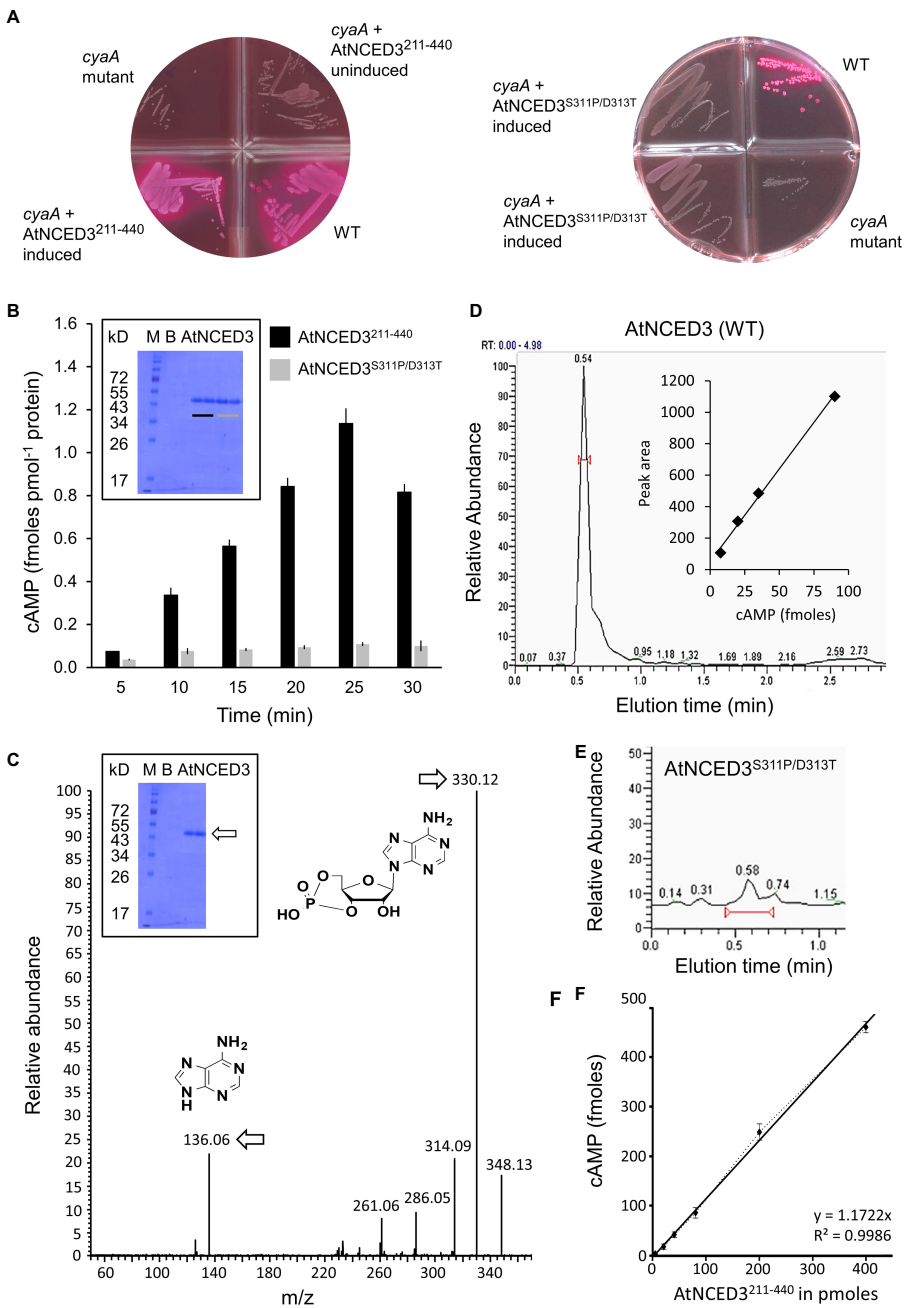
Experimental validation of AC activity *in vitro*. **(A)** Complementation of *E. coli cyaA* with AtNCED3 AC fragments AtNCED3^211-440^ (left) and AtNCED3^S311P/D313T^ (right). **(B)** Determination of cAMP generated by AtNCED3^211-440^ and AtNCED3^S311P/D313T^ using enzyme-immunoassay. Reaction mixtures contain 10 μg of AtNCED3^211-440^ or AtNCED3^S311P/D313T^, 50 mM Tris–HCl pH 8; 2 mM IBMX, 1 mM ATP, and 5 mM MnCl_2_. Measurements of three independent experiments are represented as mean±SE. The inset shows an SDS-PAGE gel of the AtNCED3^211-440^ (black bar) and AtNCED3^S311P/D313T^ (grey bar) recombinant proteins. B = blank; M = marker. **(C)** Mass spectrometric detection of cAMP. A representative ion chromatogram of cAMP showing the parent and daughter ion peaks for AtNCED3^211-440^ (see arrows). The inset shows an SDS-PAGE gel of the AtNCED3^211-440^ recombinant protein. B = blank; M = marker. Detection is based on selected reaction monitoring of cAMP by fragmenting its precursor ion at m/z 330 and yielding a product ion at m/z 136. The standard calibration curve is based on the peak areas of each calibration concentration using the extracted ion chromatogram of product ion m/z 136, and quantitation is based on the chromatographic peak areas of the samples using the extracted ion chromatogram of product ion m/z 136 (Raji and Gehring, 2017). **(D)** HPLC elution profile of cAMP generated by AtNCED3^211-440^. The calculated amount of cAMP after 25 min of enzymatic conversion of AtNCED3^211-440^ was 1.14 ± 0.1 fmoles pmol^−1^ protein (*n* = 3). The inset shows a calibration curve of the peak height plotted against fmoles of cAMP. **(E)** Cyclic AMP generated by the mutant protein (AtNCED3^S311P/D313^) shows a strongly diminished peak as compared to the AtNCED3^211-440^. **(F)** Amount of cAMP produced after 25 min as a function of the concentration of AtNCED3^211-440^. Values are means ± SD (*n* = 3).

Functional analysis of AtNCED3 AC was conducted using two methods extensively described in the Supplementary Material. The first is based on the rescue of an *E. coli* mutant (*cyaA*) deficient in its AC activity. AtNCED3^211-440^ was cloned and expressed in *E. coli* SP850 strain lacking the c*yaA* gene which prevents lactose fermentation. Due to the *cyaA* mutation, the AC-deficient *E. coli* and the uninduced transformed *E. coli* cells remain colorless when grown on MacConkey agar (Figure 2A). In contrast, the AtNCED3^211-440^ transformed *E. coli* SP850 cells, when induced with 0.5 mM IPTG, form red-colored colonies much like the WT *E. coli*, indicating that a functional AC center in the recombinant AtNCED3^211-440^ has rescued the *E. coli cyaA* mutant. Significantly, a double-mutant AtNCED3^S311P/D313T^ is unable to rescue the *E. coli cyaA* mutant (Figure 2A). S311 in position 1 of the AC motif is predicted to hydrogen bond with ATP, while D311 in position 3 is hypothesized to confer substrate specificity. This implies that AtNCED3^211-440^ contains a functional AC that can complement the *E. coli cyaA* mutant and that S311 and/or D311 is critical for catalysis of similar enzymatic centers (e.g., (Ruzvidzo et al., 2019)).

For the *in vitro* assessment of AC activity, enzyme immunoassay (Figure 2B) and mass spectrometry (LC–MS/MS) detection were performed. Both the AtNCED3^211-440^ and the AtNCED3^S311P/D313T^ double mutant were expressed in *E. coli* and affinity purified (see Supplementary Materials). Their AC activities were tested in reaction mixtures containing ATP and Mn^2+^ as the cofactor. AtNCED3^211-440^ generated cAMP in a time-dependent manner reaching a maximum of 1.16 ± 0.1 fmoles cAMP pmol^−1^ protein after 25 min, and notably, the cAMP generated by the double-mutant AtNCED3^S311P/D313T^ was ≥10 x lower (Figure 2B).

Furthermore, cAMP generation was confirmed by LC–MS/MS where the detection is based on selected reaction monitoring of cAMP by fragmenting its precursor ion at m/z 330 [M+H]^+^ and yielding a product ion at m/z 136 [M+H]^+^. The standard calibration curve is based on the peak areas of each calibration concentration using the extracted ion chromatogram of product ion m/z 136, and quantitation is based on the chromatographic peak areas of the samples using the extracted ion chromatogram of product ion m/z 136 [M+H]^+^ (Raji and Gehring, 2017). A representative ion chromatogram of cAMP showing both the parent and product ion peaks is shown in Figure 2C. AtNCED3^211-440^ generated 1.275 ± 0.1 fmoles pmol^−1^ protein of cAMP after 25 min (Figure 2D). This amount is not only consistent with the enzyme immunoassay measurements, but also comparable with other experimentally characterized plant ACs that function as moonlighting proteins, such as AtKUP7 (Al-Younis et al., 2015) and AtKUP5 (Al-Younis et al., 2018). Cyclic AMP generated by the double-mutant AtNCED3^S311P/D313^ shows a strongly reduced peak as compared to the AtNCED3^211-440^ in the LC–MS/MS detection (Figures 2D,E). This finding is entirely consistent with the identification of essential catalytic residues in the predicted AC center.

To further demonstrate the AC function of AtNCED3^211-440^, we conducted an independent set of experiment with independent batch of recombinant proteins and showed that cAMP generation increases with the amount of enzyme (Figure 2F; Supplementary Figure 2) recording a *V*_max_ of 3.0987 ± 0.08 fmoles cAMP μg protein^−1^ min^−1^ (0.0774 ± 0.0017 fmoles cAMP pmol^−1^ protein min^−1^) and a *K*_m_ of 0.851 mm, respectively (*n* = 6) (Supplementary Figure 3). The k_cat_ of the reaction *in vitro* is 1.32 × 10^−6^ s^−1^, and the k_cat_/*K*_m_ is 0.0015 M^−1^ s^−1^. The *K*_m_ of NCED3 is comparable to the human soluble AC which is reported to be 0.8 mm although its *V*_max_ is about one order of magnitude lower than that of the human soluble AC (Litvin et al., 2003). Notably, a recently characterized disease-resistant protein that is involved in ABA-mediated resistance to heat stress in *Zea mays* also has AC activity comparable to NCED3 (Yang et al., 2021).

The seemingly low *in vitro* activity of these AC centers may be attributed to their moonlighting nature. Unlike canonical stand-alone enzymes, they only assume modulatory roles regulating the function of other primary domains in complex proteins to afford localized intrinsic regulation of protein domains within micro-environments of the plant cell, such as by rapidly switching from one signaling pathway to another (Muleya et al., 2014; Irving et al., 2018). Furthermore, there is also a likelihood that components which are not present in the *in vitro* reaction mixture might significantly enhance enzymatic activity. One such component is calcium which we have found to significantly enhance NCED3 activity *in vitro* (Supplementary Figure 4). Thus, it is conceivable that cellular ion concentrations (of, e.g., calcium and bicarbonate) could regulate the AC activity of AtNCED3 (Litvin et al., 2003).

### Meta-Analysis of 9-Cis-Epoxycarotenoid Dioxygenase 3

The dioxygenase activity of NCED3 requires the binding of Fe^2+^ octahedrally to four histidine residues (Figure 1C) where the ligated oxygen is used to cleave the aromatic rings of the 9-*cis*-epoxycarotenoid substrate to produce 2-*cis*, 4-trans-xanthoxin and 12'-apo-carotenal. This constitutes the first step of ABA biosynthesis from carotenoids (Messing et al., 2010). The presence of histidines at the corresponding location in the amino acid sequence (Figure 1A) would indicate if Fe^2+^ binding and AC catalytic centers co-locate. If so, this implies that AtNCED3 has either dioxygenase or AC activity at any given time.

Indeed, the structural analysis of AtNCED3 and ZmNCED3 (PDB ID: 3NPE) showed high resemblance at their corresponding dioxygenase domains. The histidine residues of AtNCED3 are located deep into a pocket of the dioxygenase catalytic site much like in ZmNCED3 (Figure 1C). Comparison of their ribbon models also revealed that the four iron coordinating histidines occupy similar spatial arrangements as in ZmNCED3. The surface models revealed that while the AC center is located at the solvent exposed region at the entrance of the dioxygenase pocket, there is however no physical obstruction or steric hindrance of the 9-*cis*-epoxycarotenoid substrate, with or without docking of ATP at the AC catalytic center (Figure 1C).

Our sequence and structural analysis imply that both activities could occur at the same time and could be independently regulated. We therefore propose that AtNCED3 is a chloroplastic protein which acts at the intersection of two important signaling pathways involved in ABA and cAMP metabolisms. Notably, both enzymatic activities have well-documented physiological effects, including the regulation of plant osmosis, responses to salinity, energy metabolism, and light stress responses (Donaldson et al., 2004; Thomas et al., 2013; Alqurashi et al., 2016). Dual activity in moonlighting regulatory proteins is not uncommon especially in light of recent reports that have established catalytic centers of such nature (GCs and ACs) to perform a “tuning role” in complex regulatory networks, such as those described by (Irving et al., 2012; Kwezi et al., 2018).

While the biosynthesis and the molecular and biological functions of ABA are already well-understood, the same cannot be said for cAMP especially with respect to its function in the chloroplast. Experimental evidence as early as 1996 has confirmed that cAMP, but not cGMP and AMP, inhibits phosphorylation of proteins in the chloroplast especially those related to the light-harvesting chlorophyll a/b-binding protein complex (Gangwani et al., 1996). Later in 2004, cAMP was detected and quantified using a highly sensitive liquid chromatography/electrospray ionization tandem mass spectrometry method (Witters et al., 2004), which is also employed in this study. In 2005, the same group went on to demonstrate the topological AC activities in the chloroplast using cytoenzymological and immune-cytochemical approaches. They have detected AC activities in the intermembrane space and importantly also in the stroma of the chloroplast (Witters et al., 2004). Since AtNCED3 is localized in the stroma, we may have identified the molecule that is responsible, at least in part, for the reported AC activities. Furthermore, the fact that AtNCED3 is partially bound to thylakoids (Tan et al., 2003) made this argument even more convincing because it would allow cAMP, which is known to operate in a transient and temporal manner within organelle micro-environments, to selectively exert the reported stronger phosphorylation inhibitory effects on proteins in the light-harvesting complex, that are also bound to the thylakoid membranes (Gangwani et al., 1996).

### Conclusion and Outlook

Taken together, we have shown experimental evidence for what could be the first AC reported in the chloroplast of higher plants and have assigned an important moonlighting role for AtNCED3; a key enzyme involved in the synthesis of ABA. Emerging evidence has increasingly fit cAMP in the signaling pathways of ABA and ABA-dependent plant stress responses. For instance, the recently characterized disease-resistant protein RPP13-like protein 3 (ZmRPP13-LK3) not only has AC activity comparable to AtNCED3 but also is involved in ABA-mediated resistance to heat stress in maize (Yang et al., 2021). The authors showed that ZmRPP13-LK3 and cAMP are both decreased in a maize mutant that is deficient in ABA biosynthesis, viviparous-5 (vp5) and further speculated that ZmRPP13-LK3 relieves heat-induced oxidative stress through its interaction with a possible cAMP exporter ZmABC2 leading to an increase in the expression of heat-shock proteins (Yang et al., 2021). Therefore, their results implied a role for this maize AC in conferring ABA-mediated tolerance to heat. Since AtNCED3 and ZmRPP13-LK3 are, respectively, located in chloroplast and mitochondria, characterization of AC-dependent downstream molecular processes and the physiological responses may provide opportunities for organelle-level manipulations for the generation of crops with increased tolerance to abiotic stresses.

Meanwhile, in the guard cell, cAMP has long been shown to be involved in ABA-mediated K^+^ efflux through outward-rectifying channels, such as AtGORK (Jin and Wu, 1999; Lemtiri-Chlieh and Berkowitz, 2004). Salt stress increases cellular ABA but reduces K^+^ levels through the combined downregulation of guard cell-specific K^+^ influx channel AtKAT2 and upregulation of guard cell-specific K^+^ efflux channel AtGORK genes, thus leading to stomatal closure (Ache et al., 2000; Karimi et al., 2021). Since AtNCED3 is annotated as “response to osmotic stress” and “hyperosmotic salinity response” and “response to water deprivation” (Sato et al., 2018; Kalladan et al., 2019), it is conceivable that during salt stress, this gene is upregulated resulting in increased ABA and cAMP levels; the latter could then mediate ABA signaling through, e.g., the activation of ion channels. For instance, extracellular perception of ABA by AtGORK led to the direct modulation of its K^+^ conductance and this provided an alternative ABA signaling mechanism that bypasses sensing of ABA by intracellular ABA receptors PYR/PYL/RCARs (Ooi et al., 2017). Notably, AtGORK harbors a conserved cyclic nucleotide-binding domain at the cytosolic region reminiscence of that in the cyclic nucleotide-gated channels (CNGCs) (Leng et al., 2002; Talke et al., 2003). This might imply a role for cAMP in mediating the extracellular perception of ABA leading to an optimized K^+^ conductance in AtGORK. Indeed, CNGC12 is strongly induced by salt stress in guard cells and the resulting Ca^2+^ influx activated S-type anion channels to cause stomatal closure (Dietrich et al., 2020). Additionally, the intracellular ABA signaling cascades of *Arabidopsis thaliana* beginning from the intracellular ABA receptors PYL5 and PYL6, type 2C protein phosphatases PP2C (Zhang et al., 2014), to the Ca^2+^-independent and Ca^2+^-dependent protein kinases SnRK2.9 and SnRK3/CIPK, were all upregulated in guard cells during salt stress (Karimi et al., 2021).

In the roots, potassium transporters, such as AtKUP5 and AtKUP7, also contain functional AC domains that are similar in nature to AtNCED3 (Al-Younis et al., 2015; Al-Younis et al., 2018). In AtKUP5 for instance, mutation to the amino acid essential for AC activity S81P not only abolishes the AC activity but also alters the phosphorylation state (Al-Younis et al., 2018). Since this amino acid has been previously reported to be phosphorylated (Xue et al., 2013; Zhang et al., 2013), phosphorylation could both activate the AC center and modulate the K^+^ transport activity of AtKUP5. Moreover, although the K^+^ uptake is not affected by cAMP, K^+^ conductance causes cytosolic cAMP accumulation likely *via* the activation of the AC domain in AtKUP5. Thus, as a cAMP-dependent K^+^ flux sensor, cAMP generated by AtKUP5 AC in respond to its K^+^ conductance could initiate downstream signal transduction cascades acting on other cation channels, e.g., CNGCs or protein kinases which could through a feedback respond, phosphorylate AtKUP5 to fine-tune K^+^ homeostasis (Al-Younis et al., 2018). Furthermore, it has been previously demonstrated that cAMP regulates Na^+^, K^+^, and Ca^2+^ fluxes in the roots of *Arabidopsis thaliana* (Maathuis and Sanders, 2001; Ordoñez et al., 2014). Importantly, in the roots of both *Arabidopsis thaliana* and maize, ABA could regulate the activities of K^+^ channels and to some extent also K^+^ transport (Roberts and Snowman, 2000; Xu et al., 2021). Thus, ABA and cAMP elevation achieved through, e.g., NCED3, could modulate ABA-mediated signaling processes in the roots.

Since many ACs identified through this motif-based approach could, despite their low activities, affect important physiological processes (Thomas et al., 2013; Alqurashi et al., 2016; Donaldson et al., 2016), we propose that such ACs be examined from a different perspective; one that considers their low activities as an innate feature of regulatory ACs embedded within complex plant proteins. We have therefore termed these proteins as crypto-ACs. This adds to a growing list of plant proteins that are known to moonlight (Wong and Gehring, 2013; Muleya et al., 2014; Irving et al., 2018; Su et al., 2019) and where the roles of some have already been characterized and shown direct interdependence between the cyclase and other domains thereby directly affecting, e.g., ion transport (Al-Younis et al., 2015; Al-Younis et al., 2018) or enzyme activation or deactivation (Muleya et al., 2014; Wheeler et al., 2017). Our results will therefore guide future experimental work that focuses on the role of this AC in inter- and intra-molecular regulation in the chloroplast and, importantly, also its cellular and biological roles in higher plants. Future works can also employ this amino acid motif-based search strategy to identify candidate ACs in other species, including *Homo sapiens*, where structural analysis can then filter candidates for experimental studies *in vitro* and/or *in vivo*.

## Data Availability Statement

The original contributions presented in the study are included in the article/Supplementary Material, further inquiries can be directed to the corresponding author/s.

## Author Contributions

CG conceived the project. AW did the structural modeling. IA-Y, BM, KJ, and MK performed the experiments. All authors contributed to the analyses and interpretation of the data and the writing of the manuscript.

## Conflict of Interest

The authors declare that the research was conducted in the absence of any commercial or financial relationships that could be construed as a potential conflict of interest.

## Publisher’s Note

All claims expressed in this article are solely those of the authors and do not necessarily represent those of their affiliated organizations, or those of the publisher, the editors and the reviewers. Any product that may be evaluated in this article, or claim that may be made by its manufacturer, is not guaranteed or endorsed by the publisher.
