# Supplementary material for: Functional Crypto-Adenylate Cyclases Operate in Complex Plant Proteins
Source: Front Plant Sci. 2021 Aug 12;12:711749. doi: 10.3389/fpls.2021.711749 (PMC8387589; doi:10.3389/fpls.2021.711749)
Supplement: Supplementary file 1 [file Data_Sheet_1.docx]

Supplementary Material

# Supplementary Materials and Methods

## Structural analysis of AtNCED3 AC and NCED3 domains

AtNCED3 was modeled against the crystal structure of a maize viviparous14 protein (PDB ID: 3NPE) using MODELLER (ver. 9.25) (Sali and Blundell, 1993) and docking simulations of ATP to the AC center of AtNCED3 was performed using AutoDock Vina (ver. 1.1.2) (Trott and Olson, 2010). In docking simulations, all bonds in the ATP were allowed to move freely but the protein was set rigid. Docking orientations of ATP were evaluated based on a previously ascertained "correct binding pose" where adenine points towards position 1, which normally occupies the interior of the AC pocket, and phosphate points towards position 14, which normally occupies the solvent-exposed entrance area of the AC pocket (Wong et al., 2018). Docking simulations consider both spatial and charge in the vicinity of the catalytic center based on pre-determined grids that cover the entire AC center and can afford free rotation of ATP substrate which we have set prior to docking experiments. The ATP docking poses were analyzed, and all images created by UCSF Chimera (ver. 1.10.1) (Pettersen et al., 2004). Chimera was developed by the Resource for Biocomputing, Visualization, and Informatics at the University of California, San Francisco (supported by NIGMS P41-GM103311).

**1.2 Generation of recombinant AtNCED3^211-440^**

RNA was extracted from *A. thaliana* Col-0 leaf tissue using the RNeasy kit (Qiagen, Crawley, UK) and converted to cDNA using Superscript III Reverse Transcriptase according to the manufacturer’s instructions (Invitrogen, Carlsbad, CA, US). Primers designed to amplify the AC domain of AtNCED3^211-440^ (forward: 5′-ATGATAGTCGACCCGGCACA-3′ and AtNCED3^211-440^, reverse: 5′-TTAAGCATCAATCCACTTAATGTTCGA-3′). The cDNA was used as template in a PCR reaction with the AtNCED3^211-440^ AC primers and KAPA HiFi Taq Polymerase according to the manufacturer’s instructions (KAPA Biosystems, Wilmington, MA, US). Subsequently, A overhangs were added using KAPA Taq Polymerase according to the manufacturer’s instructions (KAPA Biosystems, Wilmington, MA, US) and the PCR product was cloned into the Gateway compatible pCR8 vector (Invitrogen, Carlsbad, CA, US). The AtNCED3^S311P/D313T^ double mutant was generated by site directed mutagenesis using the following primers: AtNCED3^S311P/D318T^ forward (5′- TCGCTTTAGGCTACTACGTCGTT-3′) and AtNCED3^S311P/D318T^ reverse (5’-AACGACGTAGTAGCCTAAAGCGA-3′).

The AC domain of AtNCED3, and the double mutant were recombined into the pDEST17 expression vector (Invitrogen, Carlsbad, CA, US) to create pDEST17-AtNCED3^211-440^ fusion constructs containing C-terminal His tags for affinity purification. These constructs were then transformed into *E. coli* cyaA mutants for functional complementation or *E. coli* BL21 A1 cells (Invitrogen, Carlsbad, CA, US) for recombinant protein expression. Purification of the recombinant proteins was performed under denaturing conditions using Ni-NTA agarose beads according to the manufacturer’s instructions (Qiagen, Hilden, Germany) and refolded by Fast Protein Liquid Chromatography (FPLC) using HisTrap HP Ni-NTA columns (GE Healthcare, Little Chalfont, UK) as detailed in the next section.

**1.3 Purification and refolding of recombinant AtNCED3**

The recombinant cDNA encoding *AtNCED3^211-440^* in the appropriate pDEST17-AtNCED3 ^211-440^ fusion construct was transformed into BL21 A1 *E. coli* cells (Invitrogen, Carlsbad, US) and grown in LB broth media containing 100 μg/mL ampicillin on an orbital shaker (New Brunswick Scientific, New Jersey, USA) at 200 rpm at 37°C, until the optical density (OD_600_) reached 0.6. Recombinant protein expression was induced by adding 0.2 % l-arabinose and the culture grown for a further 4 hours at 37°C. The recombinant protein was purified by preparing a cleared cell lysate under denaturing conditions essentially as described in Protocols 10 and 17 of the QIAexpressionist manual (Qiagen, Crawley, UK) but with some modifications. Firstly, a cleared cell lysate was prepared by resuspending the harvested cells in lysis buffer (100 mM NaH_2_PO_4_, 10 mM Tris-Cl, 6 M guanidine hydrochloride; pH 8) at a ratio of 1 g pellet weight to 10 mL buffer volume and mixed with on a rotary mixer for 30 min and then centrifuged at 2300 x *g* for 15 minutes at room temperature. The cleared cell lysate supernatant was collected and mixed with 1 mL 50% (w/v) Ni-NTA slurry (Qiagen, Crawley, UK) that had been pre-equilibrated with 10 mL of lysis buffer. The contents were gently mixed on a rotary mixer (Breda Scientific, Breda, Netherlands) for 30 minutes at room temperature. The lysate-resin mixture was loaded into an empty PD-10 column (Amersham Pharmacia Biotech, Little Chalfont, UK), allowed to settle and the flow through discarded. The protein bound resin was washed three times with 30 mL wash buffer (8 M urea, 100 mM NaH_2_PO_4_, 10 mM Tris-HCl; pH 6.3) then with 2 mL elution buffer (8 M urea, 100 mM NaH_2_PO_4_, 10 mM Tris-HCl; pH 5.9) and fractions collected. The recombinant protein was subjected to a second elution with 2 mL imidazole-containing elution buffer (8 M urea, 100 mM NaH_2_PO_4_, 250 mM imidazole, 10 mM Tris-HCl; pH 8). The elution fractions that contained protein were pooled then desalted and concentrated to approximately 0.5 mL using the Amicon Ultra 15 Centrifugal Filter Unit, 15 kDa NMWL according to the manufacturer’s instructions (Merck Millipore, Burlington, MA). This was diluted with 15 mL binding buffer (8 M urea, 20 mM Na_2_H_2_PO_4_, 500 mM NaCl, 100 mM sucrose, 100 mM non-detergent sulfobetaines, 0.05% polyethylene glycol, 4 mM reduced glutathione, 0.04 mM oxidized glutathione and SIGMAFAST protease inhibitor cocktail at pH 7.8). The 1 mL HisTrap HP Ni-NTA column (GE Healthcare, Little Chalfont, UK) was connected to the AKTA Fast Protein Liquid Chromatography (FPLC) (GE Healthcare, Little Chalfont, UK) and equilibrated with 10 mL binding buffer at a flow rate of 1 mL/ minute. The denatured protein was then loaded on to the column at a flow rate of 0.2 mL/ minute. Once bound, the denatured protein was refolded by a gradual linear dilution of the 8 M urea to 0 M urea in refolding buffer (20 mM Na_2_H_2_PO_4_, 500 mM NaCl, 500 mM sucrose, 100 mM non-detergent sulfobetaines, 0.05% PEG, 4 mM reduced glutathione, 0.04 mM oxidized glutathione and SIGMAFAST protease inhibitor cocktail at pH 7.8) at a flow rate of 1 mL/ minute for 50 column volumes. After renaturation, the column was washed with 10 column volumes of refolding buffer. Finally, the protein was eluted in a linear gradient for 20 column volumes with elution buffer (20 mM Na_2_H_2_PO_4_, 500 mM NaCl, 500 mM sucrose, 500 mM imidazole, 100 mM NDSB, 0.05% PEG, 4 mM reduced glutathione, 0.04 mM oxidized glutathione and SIGMAFAST protease inhibitor cocktail at pH 7.8). Fractions containing the recombinant protein were pooled then de-salted and concentrated using the Amicon Ultra 15 Centrifugal Filter Unit, 15 kDa NMWL according to the manufacturer’s instructions (Merck Millipore, Burlington, MA). The protein concentration was determined by the Bradford method (Bradford, 1976) and the recombinant protein was stored at -20°C.

# 1.4 Complementation of an AC deficient *E. coli* mutant

pDEST17-AtNCED3^211-440^ constructs were transformed into the *E. coli* *cyaA* mutant SP850 strain [lam-, el4-, relA1, spoT1, cyaA1400 (:kan),thi-1] (Shah and Peterkofsky, 1991) deficient in its AC (*cyaA*) gene. (*E. coli* Genetic Stock Center, Yale University, New Haven, CT, US, Accession Number 7200). The transformation with the plasmid was done by heat shock (2 minutes at 42°C). Bacteria with *E. coli* *cyaA* mutant strain re-grown at 37°C in Luria Broth media supplemented with 100 μg/mL ampicillin and 100 μg/mL kanamycin until they reached an OD_600_ of 0.6 and then incubated with 0.5 mM isopropyl-β-D-1-thiogalactopyranoside (IPTG) for 4 hours for transgene induction prior to streaking on MacConkey agar.

## 1.5 AC enzyme immunoassay assay and mass spectroscopic detection of cAMP

Cyclic AMP was generated from reaction mixture containing 10 μg of purified recombinant protein AtNCED3^211-440^ or the mutated protein (AtNCED3^S311P/D313T^), 50 mM Tris–HCl (pH 8.0), 2 mM isobutylxanthine (IBMX; Sigma-Aldrich, St. Louis, MO, US), 5 mM MnCl_2_ and 1 mM ATP at room temperature and in a final volume of 100 μL. The reaction was stopped by adding 10 μL of ≥ 4 mM EDTA. The cAMP produced was measured with the Biotrack enzyme immunoassay using the acetylation protocol as described by the manufacturer (GE Healthcare, Little Chalfont, UK). Cyclic AMP was also detected using liquid chromatography tandem mass spectrometry (LC-MS/MS) on an LTQ Orbitrap Velos mass spectrometer (Thermo Fisher Scientific, Waltham, MA, USA) (Raji and Gehring, 2017; Wheeler et al., 2017). Briefly, separation was achieved by a Sepax SFC Cyano column (150 × 4.6 mm x 5 μm) at ambient temperature, an isocratic mix of 10 mM ammonium acetate and acetonitrile (HPLC-MS grade, ratio: 60%/40%) and for the detection, positive ESI as ionization was used. The detection is based on selected reaction monitoring of cAMP by fragmenting its precursor ion at m/z 330 and yielding a product ion at m/z 136. The standard calibration curve will be based on the peak areas of each calibration concentration using the extracted ion chromatogram of product ion m/z 136. Quantitation is based on the chromatographic peak areas of the samples using the extracted ion chromatogram of product ion m/z 136. (Raji and Gehring, 2017). All enzymatic reactions were done alongside no-protein controls and they were also subjected to LC-MS/MS detections. Signal contributions by the controls are considered as “background” which we have subtracted to obtain the actual cAMP amounts.

**1.6 Statistical analysis**

Statistical analysis was performed using an unpaired, one-tailed Student’s *t*-test (Two-Sample Assuming Unequal Variances). Measurements are represented as mean ± SE and significance were set to a threshold of *P* < 0.05 and *n* values represent number of experimental replicates. Measurements of AtNCED3^211-440^and AtNCED3^S311P/D313T^ at each time point are compared to that of the first time point, and measurements of AtNCED3^S311P/D313T^ double mutant is also compared to that of AtNCED3^211-440^at the corresponding time points.

# Supplementary Figures and Tables

## Supplementary Figures

**Supplementary Figure 1.** AtNCED3 ATP docking clusters and data, and interpretation of the docking solutions. A total of 18 solutions across two independent simulations generated by AutoDock Vina (ver. 1.1.2) (Trott and Olson, 2010) were evaluated in terms of free energies and binding poses. Orientations and binding poses were analyzed with the UCSF Chimera (ver. 1.10.1) (Pettersen et al., 2004). Chimera is developed by the Resource for Biocomputing, Visualization, and Informatics at the University of California, San Francisco (supported by NIGMS P41-GM103311). In docking simulations, all bonds in the ATP ligand were allowed to move freely but the protein was set rigid. Docking orientations of ATP were evaluated based on a previously ascertained "correct binding pose" where the adenine of ATP points towards position 1 which normally occupies the interior of the AC pocket and, the phosphate which points towards position 14 which normally occupies the solvent-exposed entrance area of the AC pocket, respectively. Docking simulations consider both spatial and charge at the vicinity of the catalytic center based on pre-determined grids that cover the entire AC center and can afford free rotation of ATP substrate which we have set prior to docking experiments. Although varying ATP binding poses may have good binding affinities, not all docking solutions possess the "correct binding pose" that we have ascertained. Thus, we manually analyzed the binding pose of each solution and mark those with the correct binding poses as **✓** and those with incorrect binding poses as **╳** . We also consider how frequent the software finds the “correct binding pose” and found that it is 55.6% with a mean binding affinity of -4.64 ± 0.03 kcal mol^-1^.


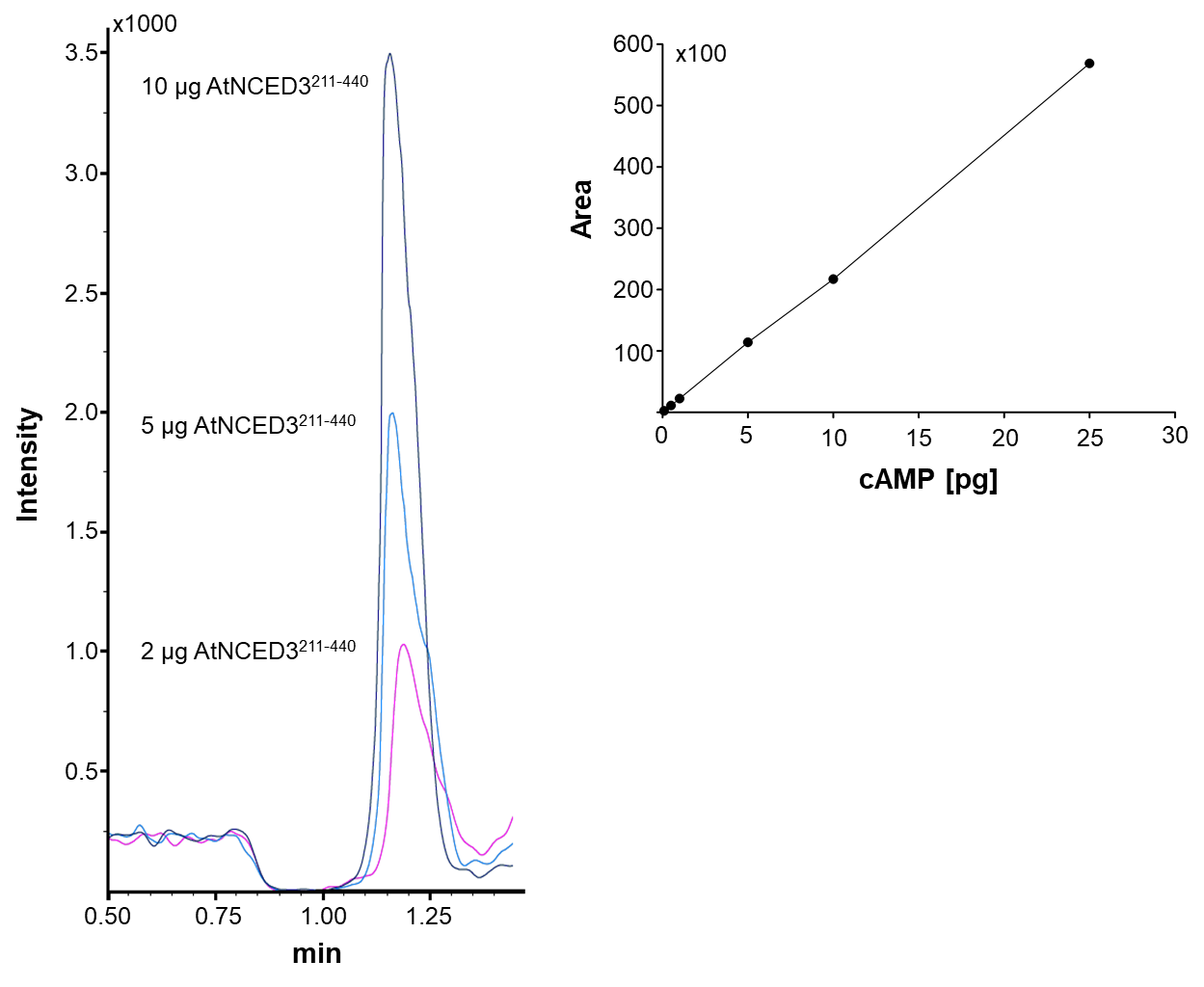


**Supplementary Figure 2.** Enzyme activity as a function of protein concentration. Representative MRM chromatograms of cAMP produced by AtNCED3^211-440^ protein at concentrations of 2, 5 and 10 µg in the presence of 50 mM Tris-HCl pH 8, 2 mM IBMX, 1 mM ATP and 5 mM MnCl_2_. Inset box shows the calibration curve for cAMP. LC-MS/MS experiments were performed using the Nexera UHPLC and LCMS-8045 integrated system (Shimadzu Corporation). The ionization source parameters were optimized in positive ESI mode using pure cAMP dissolved in HPLC-grade water (Sigma). Samples were separated using Ascentis® Express C18 HPLC Column (100 x 2.1 mm, 2.7 µm). An isocratic mix of solvent A (0.1 % (v/v) formic acid) and solvent B (100 % (v/v) methanol) (ratio: 90/10) was applied over 6 minutes with a flow rate of 0.3 mL min^-1^. The interface voltage was set at 4.0 kV for positive (ES^+^) electrospray. Data acquisition and analysis were done with LabSolutions workstation for LCMS-8045.


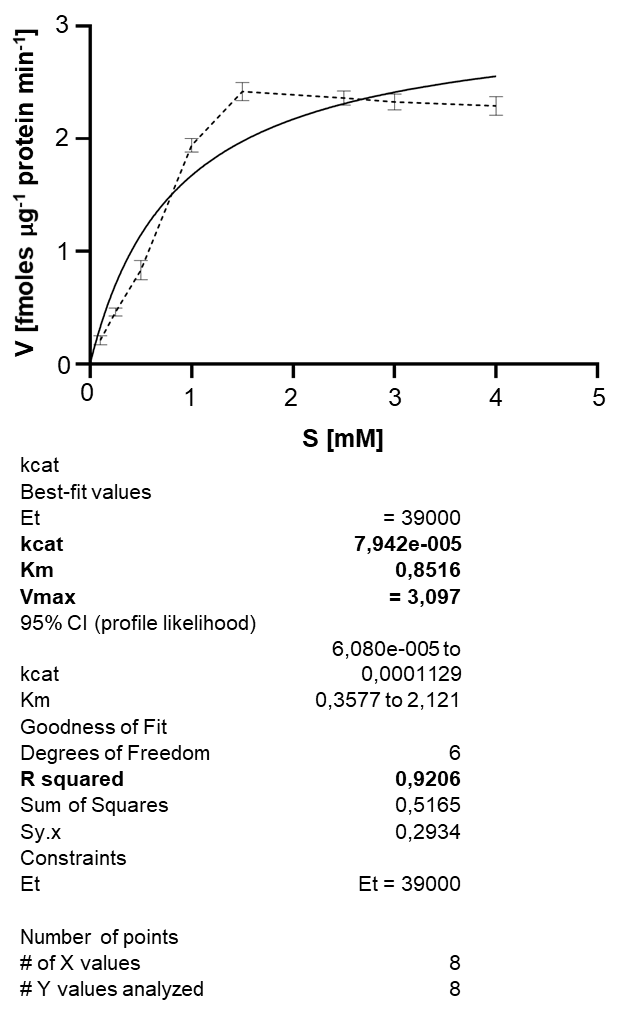


**Supplementary Figure 3.** Calculation of the *K*_m_ and *V*_max_ of AtNCED3^211-440^. Michaelis-Menten plot for adenylate cyclase activity of AtNCED3^211-440^. The *V*_max_ was 3.0987 ± 0.08 fmoles cAMP μg^-1^ protein min^-1^ (0.0774 ± 0.0017 fmoles cAMP pmol^-1^ protein min^-1^) and a *K*_m_ of 0.851 mM, respectively. Values are means ± SD (*n* = 6).


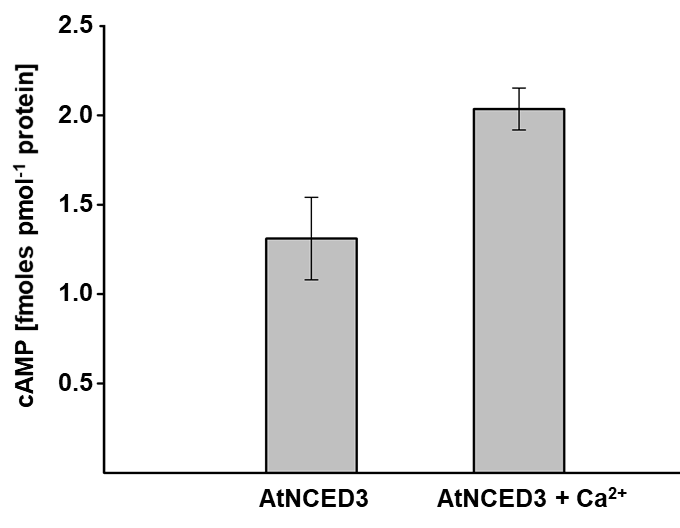


**Supplementary Figure 4.** Effect of Ca^2+^ on AC activity of AtNCED3^211-440^. Cyclic AMP generated *in vitro* by 200 pmole recombinant AtNCED3^211-440^ protein in 25 minutes in the presence of 1 µM Ca^2+^, 1 mM ATP, 5 mM MnCl_2_, 2 mM IBMX and 50 mM Tris-HCl (pH 8). Data represent means ± SD (*n* = 3).

## Supplementary Tables

**Supplementary Table 1.** *Arabidopsis thaliana* proteins that contain the AC search term [RKS][YFW][DE][VIL]X{4}[Y]X{4}[KR]X{1,3}[DE].

| **TAIR ID** | **Annotation** |
| --- | --- |
| At1g30100 ^1^ | 9-*cis*-epoxycarotenoid dioxygenase, biosynthesis of ABA - NCED5 |
| At1g47900 | Filament-like protein (DUF869) |
| At1g67120 | Homolog of the yeast MDN gene |
| At1g68110 ^3^ | CLAP, clathrin assembly protein, eap1 |
| At1g78390 ^1^  At2g22560 | 9-*cis*-epoxycarotenoid dioxygenase - NCED9  Kinase interacting (KIP1-like) family protein |
| At2g34520 ^1^ | Mitochondrial ribosomal protein S14, RPS14 |
| At3g14440 ^1,2^ | 9-*cis*-epoxycarotenoid dioxygenase - NCED3 |
| At4g18350 ^1^ | 9-*cis*-epoxycarotenoid dioxygenase - NCED2 |
| At5g59900 | Pentatricopeptide repeat (PPR) superfamily protein |
| At5g65160 | 36 carboxylate clamp (CC)-tetratricopeptide repeat (TPR) protein |
| At5g67360 | Subtilisin-like serine protein for mucilage release from seed coat |

^1^ Proteins with a function in the biosynthetic process (GO:0009058); ^2^ Annotated as having a role in the hyperosmotic salinity response ([GO:0042538](http://amigo.geneontology.org/cgi-bin/amigo/term-details.cgi?term=GO:0042538)) and the response to water deprivation ([GO:0009414)](http://amigo.geneontology.org/cgi-bin/amigo/term-details.cgi?term=GO:0009414); ^3^ Experimentally confirmed AC.

# References

Bradford, M. M. (1976). A rapid and sensitive method for the quantitation of microgram quantities of protein utilizing the principle of protein-dye binding. *Anal. Biochem.* 72, 248–254. doi:https://doi.org/10.1016/0003-2697(76)90527-3.

Pettersen, E. F., Goddard, T. D., Huang, C. C., Couch, G. S., Greenblatt, D. M., Meng, E. C., et al. (2004). UCSF Chimera - A visualization system for exploratory research and analysis. *J. Comput. Chem.* 25, 1605–1612. doi:10.1002/jcc.20084.

Raji, M., and Gehring, C. (2017). “*In vitro* assessment of guanylyl cyclase activity of plant receptor kinases,” in *Methods in Molecular Biology*, 131–140. doi:10.1007/978-1-4939-7063-6_13.

Sali, A., and Blundell, T. (1993). Comparative protein modeling by satisfaction of spatial restraints. *J Mol Biol* 234, 779–815. doi:10.1006/jmbi.1993.1626.

Shah, S., and Peterkofsky, A. (1991). Characterization and generation of *Escherichia coli* adenylate cyclase deletion mutants. *J. Bacteriol.* 173, 3238–3242. doi:10.1128/jb.173.10.3238-3242.1991.

Trott, O., and Olson, A. J. (2010). Autodock vina. *J. Comput. Chem.* 31, 2967–2970. doi:10.1002/jcc.

Wheeler, J. I., Wong, A., Marondedze, C., Groen, A. J., Kwezi, L., Freihat, L., et al. (2017). The brassinosteroid receptor BRI1 can generate cGMP enabling cGMP-dependent downstream signaling. *Plant J.* 91, 590–600. doi:10.1111/tpj.13589.

Wong, A., Tian, X., Gehring, C., and Marondedze, C. (2018). Discovery of novel functional centers with rationally designed amino acid motifs. *Comput. Struct. Biotechnol. J.* 16, 70–76. doi:10.1016/j.csbj.2018.02.007.

**
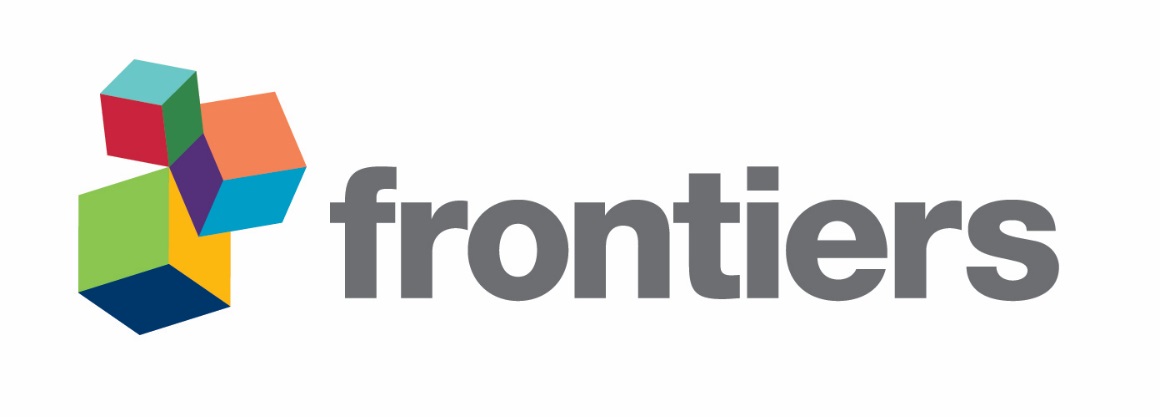
**
